# One case of myxoid glioneuronal tumour in the left frontal lobe

**DOI:** 10.1093/bjrcr/uaae014

**Published:** 2024-04-30

**Authors:** Jiayi Chu, Sheng Hu, Gangping Wang, Jibo Hu, Wenbo Xiao

**Affiliations:** Department of Radiology, The Fourth Affiliated Hospital of School of Medicine, and International School of Medicine, International Institutes of Medicine, Zhejiang University, Yiwu 322000, China; Department of Radiology, The Fourth Affiliated Hospital of School of Medicine, and International School of Medicine, International Institutes of Medicine, Zhejiang University, Yiwu 322000, China; Department of Pathology, The Fourth Affiliated Hospital of School of Medicine, and International School of Medicine, International Institutes of Medicine, Zhejiang University, Yiwu 322000, China; Department of Radiology, The Fourth Affiliated Hospital of School of Medicine, and International School of Medicine, International Institutes of Medicine, Zhejiang University, Yiwu 322000, China; Department of Radiology, The First Affiliated Hospital, College of Medicine, Zhejiang University, Hangzhou 310000, China

**Keywords:** myxoid glioneuronal tumour, tumours, 2021 CNS WHO, magnetic resonance imaging, nervous system

## Abstract

Myxoid glioneuronal tumour (MGNT), previously described as dysembryoplastic neuroepithelial tumour of the septum pellucidum, was classified as a new tumour type in the fifth edition of the WHO Central Nervous System Tumor Classification of 2021. This classification was based on its anatomical location, imaging features, and genetic characteristics. MGNTs are clinically rare and prone to misdiagnosis. In this report, we present a case of MGNT in the left frontal lobe, which was confirmed through surgical pathology.

## Introduction

Myxoid glioneuronal tumour (MGNT) is a circumscribed, low-grade glioneuronal tumour that carries a mutation at the PDGFRA codon p.K385 and is highly specific, representing a distinct central nervous system (CNS) tumour entity.^1^ Pathologically, it consists of oligodendrocyte-like tumour cells embedded in a prominent myxoid stroma. MGNT typically involves the septum pellucidum, corpus callosum, and lateral ventricles, and was previously referred to as dysembryoplastic neuroepithelial tumour (DNET) of the septum pellucidum. MGNT predominantly exhibit a cystic appearance, with most cases showing low signal intensity on T1-weighted imaging (T1WI) and high signal intensity on T2-weighted imaging (T2WI), and the majority of cases with no contrast enhancement.^2^ In our study, we present an unusual case of a patient with a lesion located in the left frontal cortical region, which exhibited a cystic-solid lesion with significant circumferential contrast enhancement. In addition, we have provided 3 follow-up images of the patient within 1 year, which demonstrate the progression of the tumour. Overall, the case we presented has specificity in terms of the location of growth and the degree of contrast enhancement, hoping to provide supplementary imaging manifestations for such rare tumours.

## Case report

**Figure 1.**
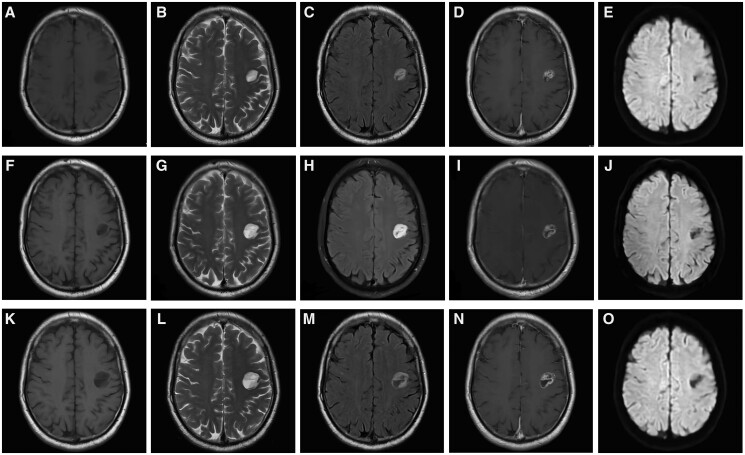
Imaging features of the myxoid glioneuronal tumour. Axial MRI showed a well-circumscribed cystic-solid lesion in the white matter area of the left frontal lobe. T1WI showed inhomogeneous low signal (A, F, K). T2WI showed inhomogeneous high signal (B, G, L). T2FLAIR shows significant high signal at the edge of the lesion (C, H, M). Contrast administration showed inhomogeneous and obvious ring-like contrast enhancement (D, I, N). DWI showed iso-low signal, with no obvious diffusion restriction (E, J, O). The lesion showed progressive enlargement during the three follow-up visits in October 2021 (A-E), February 2022 (F-J), and June 2022 (K-O). DWI, diffusion weighted imaging; MRI, magnetic resonance imaging; T1WI, T1-weighted imaging; T2WI, T2-weighted imaging.

We report a case of a patient who underwent right-sided renal cancer surgery 7 years ago, right-sided lung cancer surgery 6 years ago, and has been diagnosed with type 2 diabetes and hypertension for over 10 years. A relatively well-defined cystic appearing lesion was found in the white matter area of the left frontal lobe, measuring about 20 mm × 13 mm. The lesion showed high signal on T2WI and T2 FLAIR, low signal on T1WI, and iso-low signal on DWI which did not demonstrate any evidence of diffusion restriction. After contrast administration, the lesion demonstrates thick marginal and peripheral irregular enhancement (with a small focus of nodularity). Imaging findings suggest a high possibility of tumour, as the thicker more irregular enhancing wall suggest or lean towards a tumour rather than an abscess. The patient underwent 2 follow-up visits afterwards, and through 8 months of follow-up, we found that the lesion was gradually increasing (Figure 1).

In June 2022, the patient visited the hospital due to headache and dizziness, and the physical examination upon admission showed: body temperature was 36.8°C, respiration was 17 breaths/min, pulse was 66 beats/min, and the blood pressure was 110/59 mmHg. There was no increase or decrease in muscle tone, and muscle strength was normal, grade 5. Tumour markers: cytokeratin 19 fragment was 2.63 ng/ml (normal range <2.08 ng/ml).

**Figure 2.**
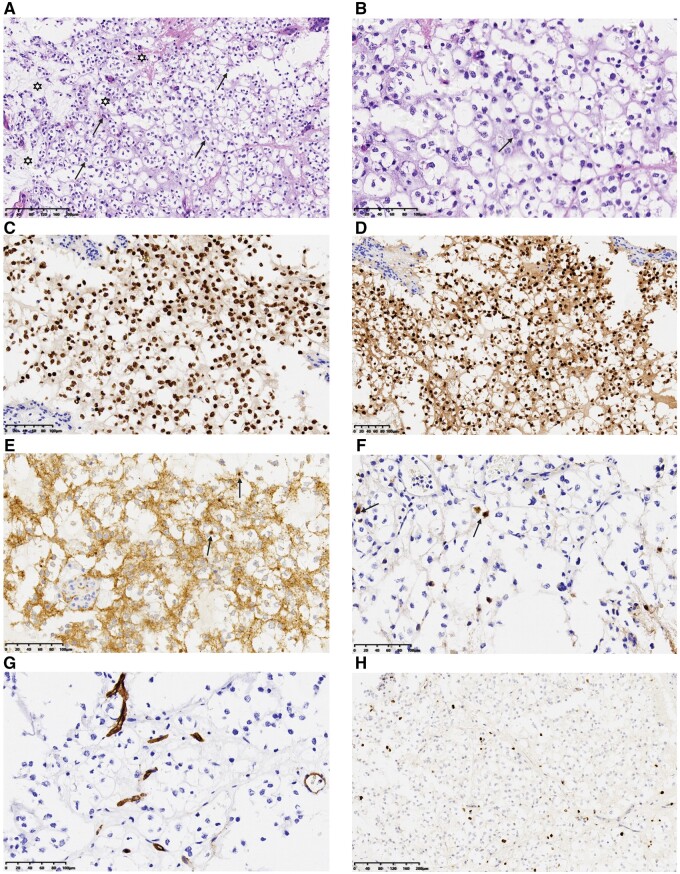
Histologic and immunohistochemical features of the myxoid glioneuronal tumour. The tumour was characterized by a proliferation of oligodendrocyte-like tumour cells (A) (black arrow) embedded in a prominent myxoid stroma (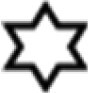), including admixed floating neurons (B) (black arrow), lacking the multinodularity with patterned mucin-rich nodules. Immunohistochemical examination showed diffuse OLIG2 (C) and SOX10 (D) nuclear positivity. There was weak granular synaptophysin staining throughout the background neuropil and highlighted the occasional floating neurons (E) (black arrow). NSE staining was only positive in rare floating neurons (F) (black arrow). CD34 immunoreactivity was limited to vascular endothelial cells only, with no CD34-positive ramified cells identified (G). The Ki-67 labelling index was uniformly low, ranging from 1% to 3% (H). NSE, non-specific esterase.

Since the tumour was located in the functional area and its increasing size, craniotomy was performed after admission to the hospital. During the operation, a round-like mass was seen at the left frontal lobe, which was grayish-yellow in colour, soft in texture, with general blood supply and clear boundary with normal brain tissue. Pathological examination of the surgical specimen suggested that it was an MGNT, WHO grade I. The tumour was characterized by a proliferation of oligodendrocyte-like tumour cells embedded in a prominent myxoid stroma, including admixed floating neurons, lack the multinodularity with patterned mucin-rich nodules that is characteristic of DNETs of the cerebral cortex. Immunohistochemical examination of the tumour shown diffuse OLIG2 and SOX10 nuclear positivity was observed in the oligodendrocyte-like tumour cells. Non-specific esterase (NSE) staining was only positive in rare floating neurons. CD34 immunoreactivity was limited to vascular endothelial cells only. The Ki-67 labelling index was uniformly low, ranging from 1% to 3% (Figure 2).

The patient experienced speech and limb movement disorders after the surgery. He received corresponding treatments including nerve nutrition and physical rehabilitation. Upon discharge, the patient's speech was somewhat fluent and experienced mild headaches. Five months later, the patient had 1 episode of seizure but has basically recovered and without any other discomfort. Subsequent imaging follow-up revealed no signs of tumour recurrence in the patient.

## Discussion

MGNT is a well-demarcated tumour of glial neuronal origin that was initially described and reported by Solomon et al^1^ in 2018. Currently, there are fewer than 100 documented cases in the literature. In the fifth edition of the WHO Classification of Tumours of the Central Nervous System (2021), MGNT has been recognized as a distinct type of “neuronal and mixed neuronal-glial tumors”, formerly referred to as DNET of the septum pellucidum.^3^ These tumours have a predilection for origin in the septum pellucidum or, less often, corpus callosum and periventricular white matter of the lateral ventricle. MGNT can be observed across a wide age range (4-65 years) with an approximately equal distribution between genders. It predominantly affects children and young adults, with the highest incidence in the second and third decades of life.^4^ Clinical manifestations vary and may include intermittent headaches, vomiting, seizures, and behavioural cognitive deficits. Some patients are incidentally diagnosed through head imaging. MGNT is characterized by a slow clinical progression and is classified as a grade I tumour according to the WHO CNS classification. It is typically curable through surgical intervention, resulting in a favourable long-term prognosis.^5^

Regarding imaging characteristics, MGNT often appears as a rounded or lobulated mass with well-circumscribed borders, ranging in size from 10 to 30 mm. On computed tomography (CT) scans, the lesions present as hypodense masses. Magnetic resonance imaging (MRI) findings of MGNT are more distinctive, typically showing a cystic appearance. These cystic areas always exhibit low signal intensity on T1-weighted images, high signal intensity on T2-weighted images, and particularly high signal intensity on FLAIR sequences (especially around cysts), and most cases do not demonstrate contrast enhancement or diffusion restriction.^6^^,^^7^ While cortical DNETs commonly present with calcification and a multinodular pattern, these features are rare in MGNT.^6^ Clinically, MGNT located in the septum pellucidum is often associated with obstructive hydrocephalus, whereas tumours centred in the corpus callosum and periventricular white matter tend not to cause hydrocephalus.

The case we are sharing also mainly presents with well-defined cyst-like changes, which are broadly similar to the common imaging manifestations of MGNT described above. It is worth noting that most previously reported cases in the literature with no contrast enhancement. However, our case presented with obvious irregular enhancement at the edge of the lesion. Furthermore, it is noteworthy that MGNT typically arises in the septum pellucidum, whereas in this particular case, the lesion was located in the left frontal cortex, further contributing to the unique imaging manifestations of MGNT. Additionally, unlike previous single-image reports, we have included 3 follow-up images showing a progressive enlargement of the lesion over an 8-month period.

Histopathologically, MGNT is characterized by the infiltration of oligodendrocyte-like tumour cells within a mucus-like interstitium. The oligodendrocyte‐like cells with monotonous round to oval nuclei, small nucleoli mucin-rich cytoplasm, and eosinophilic cytoplasm features.^8^ The presence of a prominent mucus-like interstitium infiltrated by oligodendrocyte-like cells is consistently observed in all cases, occasionally accompanied by floating neurons, neurocytic rosettes, and perivascular nerve felt structures. Calcification or acute haemorrhage are very rarely described. MGNT lacks the multi-nodularity with patterned mucin-rich nodules that is characteristic of DNET of the cerebral cortex.^9^ Rosenthal fibres, eosinophilic granular bodies, and microcalcifications typical of other low-grade neuroepithelial tumour entities are not commonly observed in MGNT. Besides, mitotic activity is typically very low or absent.^7^ Immunohistochemical analysis reveals positive expression for OLIG2, SOX10, GFAP, and MAP2. Synaptophysin positivity is observed in floating neurons and neurocytic rosettes. CD34 expression is limited to vascular endothelial cells, while proliferation indices, determined by anti-ki67 antibodies, consistently indicate low values, usually <5%.^7^

Moreover, MGNTs possess distinct molecular characteristics as they are currently the only CNS tumour entity known to harbour a PDGFRA mutation as a distinct driver gene. Notably, mutations at codon p.lys385 (p.lys385 Leu and p.lys385 Ile) have been identified as highly specific to MGNT.^1^^,^^10^ In 2018, Solomon et al reported mutations at PDGFRA codon p.K385, typically involving a dinucleotide mutation at codon 385 in the oncogene PDGFRA, resulting in the substitution of lysine with either leucine or isoleucine. Additionally, MGNT exhibits a unique DNA methylation profile with an alteration in the p.Lys385 residue, and methylation studies have confirmed the high specificity of this genetic variant.^10^ The genetic and methylation characteristics of MGNT may introduce novel ideas and approaches for nonsurgical treatments.

In differentiating MGNT from tumours originating in the septum pellucidum and ventricles, the following features are noteworthy^11^: (1) DNETs are most often located in the temporal lobe, besides, DNETs often presenting as multicystic manifestations with a high signal on T2WI. However, DNETs typically exhibit a relatively low signal on FLAIR and a high signal on the peripheral margins. Calcifications are commonly observed in DNETs but rarely seen in MGNT.^12^^,^^13^ (2) Colloid cysts, predominantly found in the third ventricle, appear as high-attenuation masses on CT and exhibit a high signal on T1WI in MRI. The signal intensity on T2WI may vary, appearing low, equal, or high.^14^ (3) Subependymomas, rare slow-growing fourth ventricular tumours typically found in middle-aged and elderly patients, may extend into the lateral ventricle. These tumours usually with no contrast enhancement and appear as solid neuroglial lesions with a high signal in the centre on FLAIR images.^15^

## Conclusion

In conclusion, we present a case of a specific MGNT located in the left frontal lobe with notable contrast enhancement. We have discussed the clinical presentation, imaging characteristics, histomorphology, and molecular features of this emerging tumour entity. It is expected that more cases accumulate and further research is conducted, it is essential to determine the appropriate pathological grading and establish clinical diagnostic guidelines for MGNT. These efforts will contribute to better serving patients with this distinct tumour type.

## Learning points

- MGNT is a rare type of brain tumour that can occur at sites other than the septum pellucidum.
- MGNT was classified as a new tumour type in the fifth edition of the WHO Central Nervous System Tumor Classification of 2021.
- A cystic appearance is characteristic of MGNT, but it can also be cystic-solid and show contrast enhancement after contrast administration.
